# *Lmo4* in the Basolateral Complex of the Amygdala Modulates Fear Learning

**DOI:** 10.1371/journal.pone.0034559

**Published:** 2012-04-03

**Authors:** Rajani Maiya, Viktor Kharazia, Amy W. Lasek, Ulrike Heberlein

**Affiliations:** 1 Ernest Gallo Clinic and Research Center, Emeryville, California, United States of America; 2 Program in Neuroscience, University of California San Francisco, San Francisco, California, United States of America; 3 Department of Anatomy, University of California San Francisco, San Francisco, California, United States of America; Radboud University, Netherlands

## Abstract

Pavlovian fear conditioning is an associative learning paradigm in which mice learn to associate a neutral conditioned stimulus with an aversive unconditioned stimulus. In this study, we demonstrate a novel role for the transcriptional regulator *Lmo4* in fear learning. LMO4 is predominantly expressed in pyramidal projection neurons of the basolateral complex of the amygdala (BLC). Mice heterozygous for a genetrap insertion in the *Lmo4* locus (*Lmo4gt/+*), which express 50% less *Lmo4* than their wild type (WT) counterparts display enhanced freezing to both the context and the cue in which they received the aversive stimulus. Small-hairpin RNA-mediated knockdown of *Lmo4* in the BLC, but not the dentate gyrus region of the hippocampus recapitulated this enhanced conditioning phenotype, suggesting an adult- and brain region-specific role for *Lmo4* in fear learning. Immunohistochemical analyses revealed an increase in the number of c-Fos positive puncta in the BLC of *Lmo4gt/+* mice in comparison to their WT counterparts after fear conditioning. Lastly, we measured anxiety-like behavior in *Lmo4gt/+* mice and in mice with BLC-specific downregulation of *Lmo4* using the elevated plus maze, open field, and light/dark box tests. Global or BLC-specific knockdown of *Lmo4* did not significantly affect anxiety-like behavior. These results suggest a selective role for LMO4 in the BLC in modulating learned but not unlearned fear.

## Introduction

Pavlovian fear conditioning (FC) is a well characterized behavioral paradigm extensively used to study learning and memory in rodents [1], [2]. In this paradigm, mice learn to associate a neutral conditioned stimulus (CS) with an aversive unconditioned stimulus (US), such that exposure to the CS by itself elicits a species-specific defensive response. Several studies implicate a central role for the basolateral complex of the amygdala (BLC, comprised of the lateral, basal, and accessory basal nuclei) in fear learning [1]. Integration of the CS and US [3] in the BLC triggers long term potentiation and activity dependent changes in gene expression which in turn result in changes in synaptic structure and function that underlie the formation of long term memories [4], [5], [6]. *De novo* gene expression has been shown to play a critical role in the formation of long term memories in a wide variety of organisms [7],[8]. Transcriptional regulators including cAMP response element binding protein (CREB) and methyl CPG binding protein 2 (MeCP2) have been shown to function in the BLC to regulate fear learning, suggesting that transcriptional regulation within the BLC may underlie aspects of aversive conditioning [9], [10].

LIM-only (LMO) proteins regulate gene transcription indirectly by interacting, via their LIM domains, with transcription co-factors and regulatory DNA–binding proteins [11], [12], [13]. An unbiased genetic screen in *Drosophila* conducted in our laboratory identified the LIM-only (*dLmo*) gene as an important regulator of cocaine sensitivity [14]. Mammalian genomes encode four LMO genes, LMO1–4. Recent findings from our laboratory implicate *Lmo4* and its transcriptional targets in the nucleus accumbens in cocaine-induced behavioral plasticity [15], [16]. *Lmo4* is also highly expressed in brain structures involved in fear learning including the BLC, hippocampus, and prefrontal cortex (PFC) [11], [15], [17], [18]. *Lmo4* levels in the brain are dynamically regulated in a circadian-dependent manner as well as by sleep deprivation [19], [20]. Mammalian homologs of dLMO have been shown to form large multimeric protein complexes that are hypothesized to repress transcription [21], [22], [23]. However, recently it has been shown that LMO4 can also activate transcription in a calcium dependent manner [24]. LMO4 could therefore impact synaptic plasticity by regulating gene transcription in an activity-dependent manner. These observations, together with the enriched expression of *Lmo4* in the fear circuit led us to hypothesize that *Lmo4* might play a role in fear learning.

Results outlined in this study highlight a novel role for the transcriptional regulator LMO4 in fear learning. LMO4 is highly expressed in the pyramidal projection neurons of the BLC. Global or adult- and BLC-specific knockdown of *Lmo4* enhanced freezing to both the context and the cue in a FC paradigm. In addition, *Lmo4gt/+* mice displayed enhanced neuronal activation after fear conditioning, which may explain the enhanced freezing observed in these mice. In addition, reduction of *Lmo4* levels globally or in the BLC did not affect unlearned or innate fear, suggesting a specific role for *Lmo4* in the BLC in modulating learned fear.

## Materials and Methods

### Ethic statement

All animal protocols were approved by the Ernest Gallo Clinic and Research Center (EGCRC) Institutional Animal Care and Use Committee (approval number 09.11.198).

### Subjects

The generation and characterization of mice with a genetrap insertion in exon 4 of *Lmo4* (designated as *Lmo4gt*) have been described previously [15]. 8–10 week old male heterozygous *Lmo4gt* (*Lmo4gt/+*) mice that were backcrossed to C57BL6/J mice for 8–9 generations and their WT littermates were used in this study. 8–12 week old C57BL6/J mice were used for stereotaxic surgeries. Mice were group housed until surgery after which they were individually housed. All animals used in the study were on a 12-hour light/dark cycle and provided with food and water ad lib.

### Antibodies

Goat anti-LMO4 antibody and rabbit anti-c-Fos antibody were from Santa Cruz biotechnology (Santa Cruz, CA). Mouse anti-CamKII-α antibody was from Upstate (Lake Placid, NY). Mouse anti-GFP monoclonal antibody (3E6) was purchased from Invitrogen (Carlsbad, CA). Monoclonal anti-calretinin antibody was from Millipore (Billerica, MA). Monoclonal anti-parvalbumin and anti-calbindin antibodies were from Sigma (St. Louis, MO).

### Lentiviral shRNA constructs

The design and cloning of small hairpin RNA's against *Lmo4* (designated as shLmo4) as well as control scrambled shRNA (designated as shScr) that does not target any gene in the mouse genome have been described [15], [25].

### Stereotaxic surgery

8–12 week old male C57BL6/J mice were infused bilaterally with lentivirus as described previously [15], [26]. Viral titers were in the range of 1×10^7^–10^8^ pg/ml. 1 µl of virus was infused into the BLC using a Hamilton syringe. The coordinates for BLC were A/P = −1.6, M/L = ±3.1, D/V = −4.8 [27]. The coordinates for the dentate gyrus (DG) sub region of the hippocampus were A/P = −1.9, M/L = ±2.1, and D/V = −2.1. Mice were injected bilaterally (both BLC and DG) and allowed to recover for 2 weeks before being subjected to behavioral testing.

### Laser capture microdissection (LCM)

Mice were infected with shLmo4 or shScr virus and allowed to recover for 3 weeks. Mice were then euthanized with CO_2_ and their brains were frozen in −50°C isopentane. 30 µm sections were collected on to membrane slides (Leica microsystems). The slides were then dehydrated with graded alcohol and xylene. Infected cells were visualized by GFP and captured using a Leica laser capture device (Leica microsystems). RNA was isolated from the collected tissue using the RNeasy kit from Qiagen (Valenica, CA). Complementary DNA (cDNA, from approximately 300–400 ng of RNA) was synthesized using reverse transcription reagents from Applied Biosystems (Foster city, CA). Following synthesis, cDNA was diluted 1∶5 in water. TaqMan qPCR was performed using standard thermal cycling conditions on an ABI PRISM 7900 Sequence Detection System (Applied Biosystems). The sequences of the mouse *Lmo4* probe and primers are published [15]. Mouse *Gapdh* probe and primers (from Applied Biosystems) were used as controls.

### Immunohistochemistry

#### Verification of placements

For GFP immunostaining to verify placements, mice were anesthetized by injecting Euthasol and perfused transcardially with 0.9% saline before perfusion with 4.0% PFA in PBS for 5 min. Brains were removed, fixed in 4% PFA overnight at 4°C and transferred to 30% sucrose solution at 4°C where they remained for 1–2 days until they were sectioned. Brains were mounted using Tissue-Tek OCT (Ted Pella Inc., Redding, CA) and 50 µm free-floating sections were cut using a cryostat (Microm, Thermo Fisher Scientific, Waltham, MA). The sections were then were pretreated with 3% H_2_O_2_ for 10 minutes followed by 50% ethanol twice for 10 minutes each. Sections were blocked with 10% normal donkey serum (NDS, Jackson Immunoresearch, West Grove, PA) for 10 minutes and incubated with mouse anti-GFP monoclonal antibody diluted 1∶1500 in PBS with 0.1% Triton-X-100 for 48 hours. Sections were washed three times for 5 minutes with PBS and then incubated with 2% NDS for 10 minutes. Biotin-conjugated donkey anti-mouse secondary antibody (diluted 1∶250; Jackson Immunoresearch, West Grove, PA, USA) was incubated with sections for 2–3 hours, followed by ExtrAvidin (1∶2500; Sigma) for 1–2 hours. Diaminobenzidine was used for brown color detection of the GFP immunostaining. Sections were washed with PBS, mounted on gelatin-coated slides and dried. Slides were stained with cresyl violet according to standard protocols.

#### Immunostaining for LMO4, GFP, calbindin, calretinin, parvalbumin, and c-Fos

For immunofluorescent detection of LMO4, GFP, calbindin, calretinin, parvalbumin, and c-Fos, sections were generated and treated as above, except fluorescently labeled secondary antibodies were used. Goat anti-LMO4 antibody was diluted 1∶500. Mouse anti-GFP was diluted 1∶1000. Rabbit anti-c-Fos antibody was diluted to 1∶1000. Mouse anti-calbindin, anti-calretinin, and anti-parvalbumin antibodies were diluted 1∶1000. Secondary antibodies were Alexa fluor-conjugated donkey anti-goat, donkey anti-mouse, and donkey anti-rabbit (Invitrogen, Carlsbad, CA), all diluted 1∶300. After staining, sections were mounted on slides with Vectashield fluorescent mounting medium containing DAPI (Vector Laboratories, Burlingame, CA, USA). Images were acquired using Zeiss LSM 510 META laser confocal microscope (Zeiss MicroImaging, Thornwood, NY) using factory recommended configurations.

Image J was used to quantify c-Fos positive puncta. Images were acquired at 10× magnification. For consistency in quantification across samples, the boundary of the amygdala was set along the antero-posterior axis from −1.4 to −2.3. The number of Fos-positive puncta/area was quantified by an observer blind to the experimental condition and genotype.

#### LMO4 and CaMKII-α double staining

To determine co-localization of LMO4 with CaMKII-α, mice were perfused with 4% paraformaldehyde in phosphate buffer; brains were postfixed for 4 hours in the same fixative, rinsed and then immersed in 30% sucrose for 48 hours. Frozen 16 µm-thick coronal sections were cut on a cryostat (Microm, Thermo Fisher Scientific, Waltham, MA) and collected on adhesive slides (Thermo Fisher Scientific). Slides with tissues were dried for 30 minutes at 37°C, and processed for heat-induced epitope retrieval (HIER) using an unmasking buffer (pH 6.0, H-3300, Vector Labs, Burlingame, CA) and a pressure chamber with the temperature set at 115°C for 30 seconds (DS2002, Biocare Medical, Concord CA). Slides were rinsed in PBS and incubated in 0.05% Trypsin (Sigma-Aldrich) in 1% normal donkey serum (NDS, Jackson Immunoresearch, West Grove, PA) in PBS at 37°C for 20 minutes, rinsed 5 times in PBS, incubated in 10% NDS for 30 minutes and incubated overnight in the mixture of primary antibodies: mouse monoclonal anti-CaMKII-α (#05-532, 1∶500, Upstate, Millipore, Billerica, MA); and goat polyclonal anti-LMO4 (C-15, 1∶200; Santa Cruz Biotechnology, Santa Cruz, CA) at the room temperature. Next day slides were rinsed in PBS incubated in 2% NDS for 10 minutes and incubated in the mixture of secondary antibodies: Alexa Fluor 488-labeled donkey anti-mouse, and Alexa Fluor 594-labeled donkey anti-goat (1∶250, Invitrogen, Carlsbad, CA) for 4 hours.

#### C-Fos and CaMKII-α double staining

Because the above HIER pre-treatment had a suppressing effect on the c-Fos antigen but was necessary for CaMKII-αstaining, we performed a sequential immunostaining procedure in which c-Fos staining was done prior to HIER and CaMKII-α staining. The anti-c-Fos primary rabbit polyclonal antibody (1∶800) was applied first using a conventional immunostaining protocol (with no HIER, and no trypsin pre-treatments) overnight, followed by rinses and 4-hour incubation in secondary Alexa Fluor 594-labeled donkey anti-rabbit. Sections were then rinsed in PBS, incubated in 4% in PFA for 45 minutes (to retain the c-Fos immunostaining) followed by 3 rinses in PBS for 5 minutes each after which HIER was applied. The trypsin step was omitted to avoid loss of c-Fos immunostaining. After blocking in 10% NDS the tissue was incubated in anti-CaMKII-α primary antibody overnight and followed by secondary Alexa Fluor 488-labeled donkey anti-mouse. To ensure that PFA-HIER treatment did not cause loss of c-Fos staining, we compared c-Fos staining before and after this step. Control experiment also included omitting primary antibodies in which no specific staining was observed. Sections were briefly air dried and coverslipped using Vectashield mounting media (Vector Labs). Images were acquired using Zeiss LSM 510 META laser confocal microscope (Zeiss MicroImaging, Thornwood, NY) using 40×/NA1.3 oil immersion lens and factory recommended configurations. Short stacks of confocal images from BLA were collected within 5–7 µm's from the tissue surface, where the antibody penetration was optimal.

### Behavioral analyses

Before testing all mice were habituated to the behavioral room for 1 hour. All behavioral testing was done at the same time of day.

#### Fear conditioning

Mice were subjected to FC using three tone-shock pairings. FC was performed in “Freeze Monitor" chambers (San Diego Instruments, San Diego, CA) that were equipped with infrared photobeams, which allowed for high resolution scoring of freezing behavior. The amount of time mice spent freezing was calculated from the beam-break data [28]. Mice were placed in the FC chambers and allowed to habituate for 2 minutes, following which they were presented with an 80-decibel tone for 30 second duration. The tone co-terminated with a 0.5 mA foot shock, which lasted for 2 seconds. Mice were subjected to two more CS/US presentations with an inter-trial interval (ITI) of 60 seconds. Beam breaks were recorded during the entire conditioning session to determine baseline (first two minutes) as well as post shock freezing. Mice were removed from the chambers 30 seconds after the delivery of the last foot shock and returned to their home cages. The chambers were cleaned between subjects using 70% ethanol. Contextual fear conditioning was analyzed 24 hours later by placing mice in the FC chambers for 3 minutes. Freezing during the entire 3 minute duration was recorded. 2 hours after contextual FC, freezing to the cue was assessed in an altered context. The context was altered by covering the walls of the chamber with paper and changing the texture of the floor. Additionally, the chambers were cleaned with a eucalyptus-scented cleaner. Mice were placed back in the chamber and subjected to 3 tone presentations, each of 30-second duration with varying ITIs. Freezing to the tone was recorded. For the “no conditioning" control group mice were placed in the FC chambers for 5 minutes but not subject to tone-shock pairings.

#### Elevated plus Maze

The elevated plus maze consisted of two open and two closed arms perpendicular to each other. The maze was elevated approximately 40 cm above the ground. The open arms measured 70 cm long×9 cm wide. The closed arms were identical to the open arms but were enclosed by a 12 cm high wall. Mice were placed in the center of the maze facing the open arms and allowed to explore the maze for 5 minutes. The entire session was recorded using a video camera and the results were scored manually by an observer blind to the genotype and experimental conditions. Parameters measured included number of open arm entries as well as the amount of time spent in open arms.

#### Light/Dark Box

The light/dark box apparatus consisted of an automated activity monitor chamber that was fitted with a light/dark box insert (ENV-510, Med Associates, St. Albans, VT). The light and dark compartments each measured 24 cm×28 cm×25 cm and were enclosed in a sound attenuating chamber. The light side of the compartment was brightly lit (100 Lux). Mice were placed in the lit chamber facing the dark compartment and their activity was recorded for 5 minutes using a photobeam-based tracking system. Time spent in each of the compartments was recorded.

#### Open field test

Mice were placed in Plexiglas locomotor activity chambers (43 cm×43 cm; MED Associates, St. Albans, VT), located in sound-attenuating cubicles equipped with a 2.8 W house light and exhaust fans that mask external noise. The chambers contain two sets of 16 pulse-modulated infrared photobeams on opposite walls to record *x*, *y* ambulatory movements, and are computer interfaced for data sampling at 100 ms resolution. Mice were placed in the chambers for 5 minutes and the amount of time they spent at the center and the periphery of the chamber was recorded. The same set of mice was used in all three behavioral paradigms measuring anxiety. The elevated plus maze test was performed first followed by the light/dark box and the open field test. Mice were allowed 1 week to recover between tests.

### Statistical analysis

Two- way repeated measures (RM) analysis of variance (ANOVA) with genotype×test (baseline, context, and cue) followed by Neumann-Keul's posthoc analyses was used to analyze fear conditioning data. Two-way ANOVA (genotype×session) followed by Bonferroni posthoc test was used for analysis of immunohistochemistry results. Student's t-test was used to analyze results from behavioral paradigms used to measure anxiety-like behavior and to analyze quantitative PCR data. All statistical analyses were performed using Sigmastat and Graph pad prism software.

## Results

**Figure 1:**
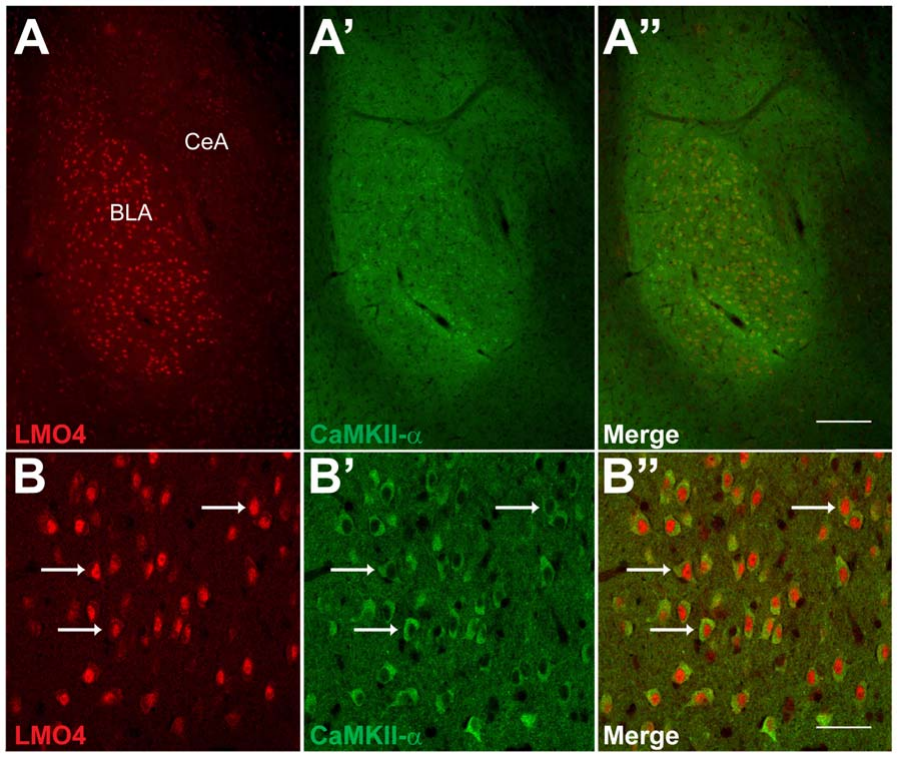
Co-localization of LMO4 and CaMKII- α in the BLC. Top panels: Dual-channel immunofluorescence images show high levels of expression for both LMO4 (A) and CaMKII-α (A′) in the BLC. Scale bar: 200 µm. Bottom panels: strong co-localization of both markers is evident in numerous BLC neurons (arrows, B, B′, and B″), with the LMO4-postive nuclei and CaMKII-α-positive perikarya. Scale bar: 50 µm.

LMO4 is highly expressed in the BLC (Figure 1). To determine the cell types in which LMO4 is expressed, we first examined co-localization of LMO4 protein with the CamKII-α subunit, a marker for pyramidal projection neurons in the BLC [29]. Our results indicate that LMO4 expression in the BLC overlaps extensively with that of CaMKII-α, with LMO4 expression localized to the nucleus and CamKII-α expression in the perikarya (Figure 1). We also examined co-localization of LMO4 with markers for three of the major interneuron populations in the BLC, namely parvalbumin, calbindin, and calretinin. LMO4 expression did not co-localize with any of these interneuron markers (Supplementary Figure S1). Taken together, these results suggest that LMO4 is predominantly expressed in pyramidal projection neurons of the BLC.

**Figure 2:**
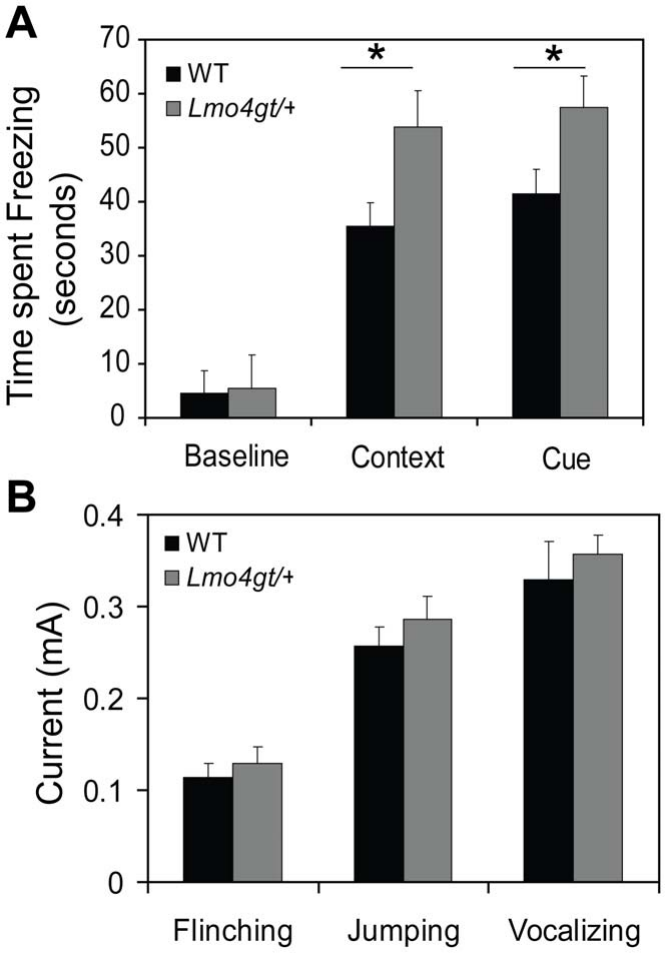
*Lmo4gt/+* mice display enhanced freezing to the context and the cue. *Lmo4gt/+* mice were subjected to FC using three tone shock pairings. Time spent freezing to the context and the cue was determined. A) *Lmo4gt/+* mice froze significantly more to both the context and the cue. Genotypic differences in baseline freezing were not observed. B) Footshock sensitivity was examined in WT and *Lmo4gt/+* mice. No significant differences were observed in current intensity required to elicit flinching, jumping, and vocalizing response between the two genotypes. Data are presented as mean ± SEM. *, p<0.05. n = 16/group for panel A, n = 7/group for panel B.

Since the function of pyramidal projection neurons is critically involved in FC [5], we analyzed this behavior in heterozygous *Lmo4gt/+* mice which express 50% less *Lmo4* than their WT counterparts [15]. *Lmo4gt/+* mice and their WT littermates were subjected to FC using three tone/footshock pairings. Two-way RM ANOVA indicated a significant main effect of genotype [F_(1, 60)_ = 10.726, p<0.005], test [F_(2, 60)_ = 158.404, p<0.001] and genotype×test interaction [F_(2, 60)_ = 5.985, p<0.005]. Neuman-Keul's post-hoc test revealed that *Lmo4gt/+* mice freeze significantly more to both the context (p<0.001) and the cue (p = 0.001) (Figure 2A) associated with the footshock. No differences were observed in postshock freezing (WT = 38.5±4.09 seconds, *Lmo4gt/+* = 46.44±5.4 seconds, p = 0.25, Student's t-test) between the genotypes. We also compared footshock sensitivity of WT and *Lmo4gt/+* mice. No genotypic differences were found in the current amplitude required to elicit flinching, jumping, and vocalizing responses (Figure 2B). These results suggest that the differences in freezing observed between the two genotypes are not due to altered perception of the US.

**Figure 3:**
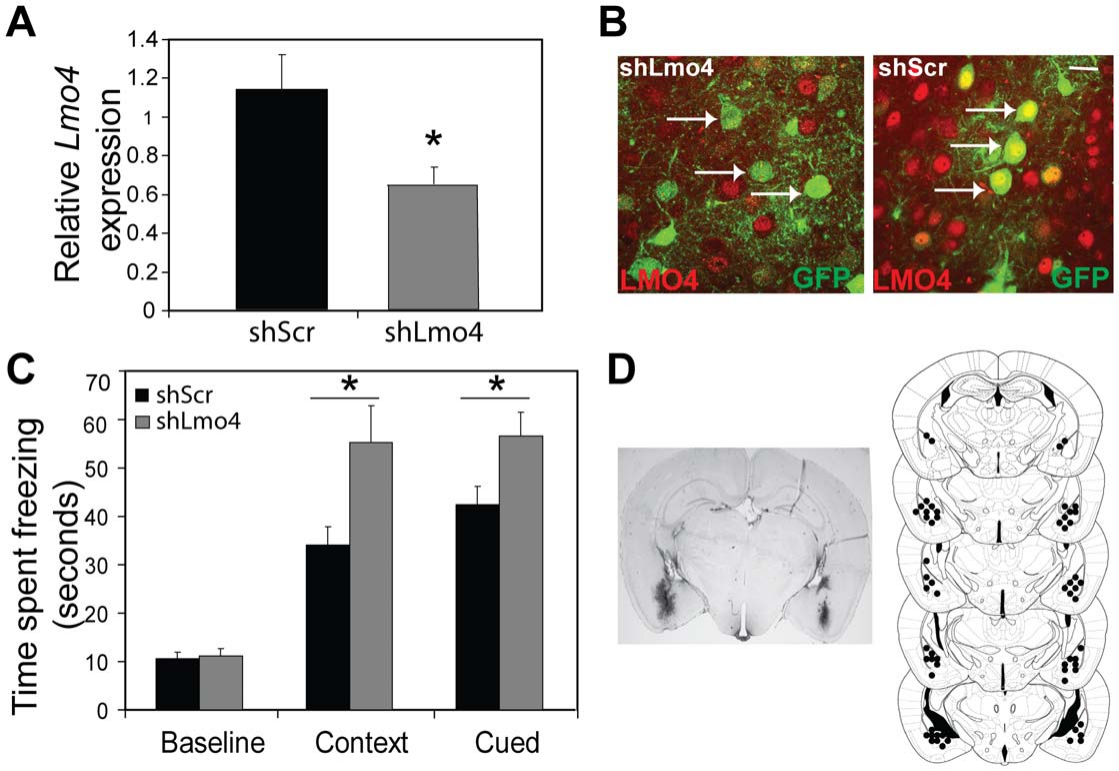
Knockdown of *Lmo4* in the BLC leads to enhanced freezing to the context and the cue. A) Quantification of knockdown of *Lmo4* (RNA) expression in the BLC. *Lmo4* levels were quantified in laser captured tissue from sh*Lmo4* and shScr infected mice by QPCR. *Lmo4* levels were significantly decreased (by 46%) in sh*Lmo4* infected cells compared to the shScr control. *, p<0.05, n = 6 (shLmo4) and 10 (shscr). B) Immunohistochemical analyses revealed a robust decrease in LMO4 expression in GFP positive/infected cells (arrows point to infected neurons, right panel) from shLmo4-injected mice but not from shScr-injected mice (arrows point to infected neurons, left panel). Co-localization of LMO4 and GFP observed as yellow overlap in nuclei can be observed in shScr- but not shLmo4-infected neurons. Scale bar: 50 µm B) Knockdown of *Lmo4* expression in the BLC results in increased freezing to both the context and the cue. No differences in baseline freezing were observed between the two different shRNA's. Data are represented as mean ± SEM. n = 10–12/group. C) Left panel: Representative image of viral infection is shown. Viral infection was visualized by staining for GFP. Right panel: Serial reconstruction of injection sites is shown.

To determine if there is an adult- and brain-region specific role for *Lmo4* in FC, short-hairpin RNAs (shRNA) were used to down-regulate *Lmo4* expression in specific brain regions. ShRNAs directed against a discrete region of *Lmo4* were generated and cloned into a GFP-expressing lentiviral vector as described [15]. A virus expressing scrambled shRNA (shScr) that does not target any gene in the mouse genome was used as a negative control. We first targeted the BLC because of the high levels of LMO4 expression in the BLC and the critical role it plays in fear learning. Quantitative PCR performed using RNA extracted from laser captured tissue from shLmo4- and shScr-infected mice revealed significant (46%) knockdown of *Lmo4* expression in the BLC of mice injected with shLmo4 (p<0.05, student's t-test) in comparison to shScr infected mice (Figure 3A). We also examined LMO4 expression in shLmo4 and shScr-infected mice. While we observed robust expression of LMO4 in GFP-positive (infected) neurons from shScr infected mice (arrows, left panel Figure 3B), we did not observe LMO4 expression in GFP-positive neurons in shLmo4-injected mice (arrows, right panel, Figure 3B). Behavioral analyses indicated that knockdown (bilateral) of *Lmo4* in the BLC recapitulated the phenotype observed in *Lmo4gt/+* mice, and results in enhanced freezing to both the context and the cue (Figure 3C). Two-way RM ANOVA revealed a significant main effect of shRNA [F_(1, 40)_ = 0.66, p<0.01], test [F_(2, 40)_ = 70.35, p<0.001], and shRNA×test interaction [F_(2, 40)_ = 3.984, p<0.026]. Further, Neumann-Keuls post hoc analysis indicated that mice injected with shLmo4 froze significantly more to both the context (p<0.001) and the cue (p<0.05) in comparison to mice injected with shScr. Differences were not observed in post shock freezing between shScr and shLmo4 injected mice (shLmo4 = 30.7±5.77 seconds, shScr = 24.5±3.4 seconds, p = 0.35, student's t-test).

**Figure 4:**
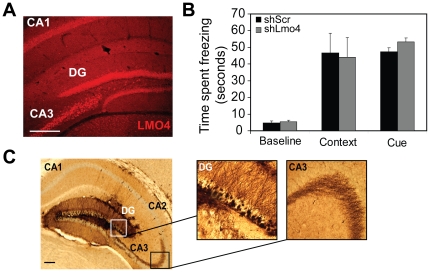
Knockdown of *Lmo4* in the DG does not affect freezing to the context or the cue. Freezing responses were measured after knockdown of *Lmo4* expression in the DG. A) LMO4 (red) is highly expressed in the DG, and CA1 and CA3 sub regions of the hippocampus. Scale bar: 200 µm. B) Knockdown of *Lmo4* in the DG sub-region of the hippocampus does not significantly affect freezing behavior to either the context or the cue. Baseline freezing was also not affected. Mean ± SEM are depicted. N = 6/group. C) Right Panel: Image depicting area of infection in the DG. Scale bar: 200 µm. Left panels: Higher resolution image of the boxed areas reveal GFP staining in the granule cells as well as in the mossy fiber projections to the CA3 region.

The hippocampus is critically involved in contextual fear learning and *Lmo4* is expressed in the CA1, CA3, and DG regions (Figure 4A) of the hippocampus [15]. Several reports suggest an important role for the DG in contextual FC [30], [31]. Hence, we decided to examine the effects of *Lmo4* downregulation in the DG on both contextual and cued FC. Knockdown of *Lmo4* in the DG did not affect freezing to either the context or the cue (Figure 4B). Two-way RM ANOVA revealed a main effect of test [F_(2, 18)_ = 27.23, p<0.001], but no effect of shRNA [F_(1,18)_ = 0.0398, p>0.05] or test×shRNA interaction [F_(2, 18)_ = 0.2, p>0.05]. Immunohistochemical analyses conducted after behavioral studies revealed GFP staining in granule cells throughout the DG as well as in the mossy fiber projections to the CA3 (Figure 4C).

**Figure 5:**
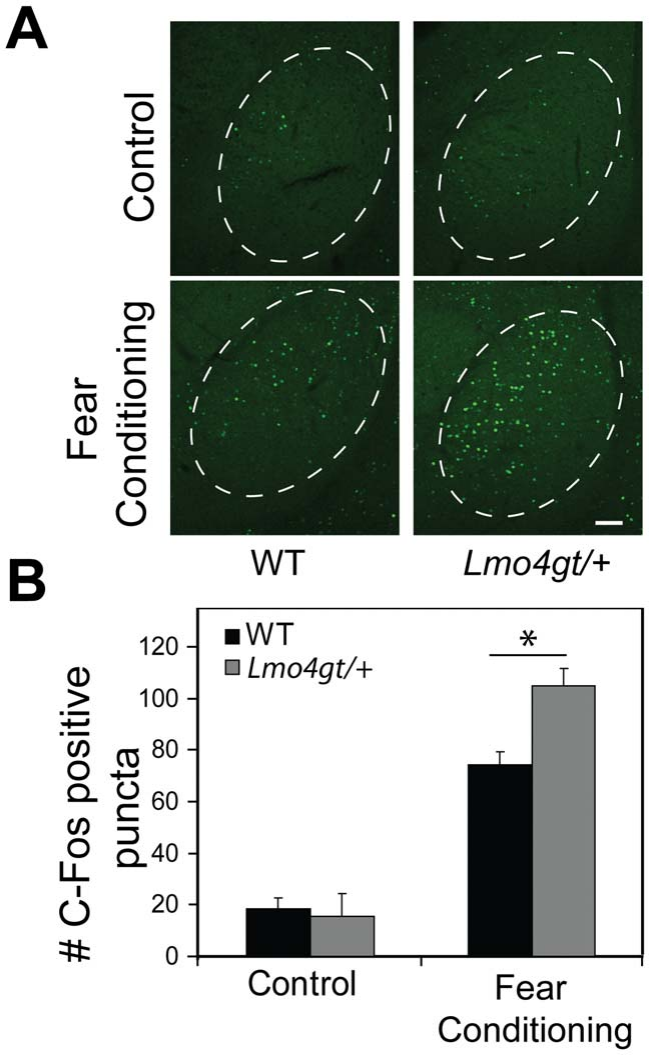
*Lmo4gt/+* mice display increased neuronal activation after FC. Neuronal activation in the BLC was assessed 2 hours after FC in both WT and *Lmo4gt/+* mice by measuring c-Fos positive puncta in the BLC. Control mice were exposed to the FC chambers but not subjected to tone/shock pairings. A) Genotypic differences in c-Fos staining were not observed in the BLC under control conditions. After FC, *Lmo4gt/+* mice displayed significantly more c-Fos positive puncta than their WT counterparts. Scale bar: 200 µm. B) Quantification of results in panel A. Data are presented as mean ± SEM. *, p<0.05. n = 3–5/group.

Integration of the CS and US in the BLC after FC initiates a cascade of signaling events that culminate with the expression of immediate-early genes such as c-Fos [4]. To determine whether downregulation of *Lmo4* in pyramidal neurons of the BLC alters neuronal activation, we examined c-Fos activation in the BLC of WT and *Lmo4gt/+* mice 2 hours after subjecting mice to fear conditioning. *Lmo4gt/+* mice displayed significantly enhanced c-Fos immunoreactivity in comparison to their WT controls (Figure 5A and B). Two way ANOVA indicated a significant main effect of test [F _(1, 66)_ = 166.69; p<0.0001], and genotype×test interaction [F _(1, 66)_ = 5.30; p = 0.025]. Bonferroni post hoc test indicated that the number of c-Fos positive puncta was significantly higher in *Lmo4gt/+* mice than in WT littermates (p<0.01) following conditioning. No differences were observed in number of c-Fos positive puncta in control WT and *Lmo4gt/+* mice that were exposed to the chambers but not subjected to CS/US pairings (p>0.05). These results suggest that reducing *Lmo4* levels in the pyramidal projection neurons leads to enhanced neuronal activation during FC and may underlie the increased freezing response observed in these mice (Figure 5A and B). To determine whether reducing *Lmo4* levels alters the recruitment of pyramidal and/or local circuit neurons in fear memory formation, we examined co-localization of c-Fos with CaMKII-α after fear conditioning in WT and *Lmo4gt/+* mice (Supplementary Figure S2). We examined 140–160 c-Fos positive cells/genotype from WT and *Lmo4gt/+* mice (n = 2 mice/genotype) and found that approximately 90% of c-Fos positive cells also stained for CaMKII-α in both genotypes (arrows Supplementary Figure S2A, top and bottom panels). Approximately 10% of c-Fos positive cells in both genotypes did not stain for CaMKII-α and are likely local interneurons (arrow, Supplementary Figure S2B). These results suggest that pyramidal projection neurons are predominantly activated in the BLC by fear learning and that reduction in *Lmo4* expression does not change the balance between excitatory and inhibitory cells in the BLC FC circuit.

The BLC is also important in modulation of unlearned fear or anxiety [32], [33]. To determine whether *Lmo4* has a role in learned as well as unlearned (innate) fear, we compared anxiety-like behavior in WT and *Lmo4gt/+* mice as well as mice in which *Lmo4* levels were down-regulated in the BLC using shRNA. We subjected mice to behavioral paradigms designed to measure anxiety-like behavior: the elevated plus maze, the open field test, and the light/dark box test. Analysis in the elevated plus maze revealed no differences in the number of open (p = 0.84, Student's t-test) and closed arm (p = 0.52) entries made by WT and *Lmo4gt/+* mice. Significant differences were also not observed in % open arm entries (p = 0.93) or in % time spent in open arms (p = 0.71) in shLmo4 and shScr injected mice (Table 1). We next subjected mice to the light/dark box test (Table 1). No differences were observed in the % time spent in the lit compartment between WT and *Lmo4gt/+* mice (p = 0.6) or ShLmo4 and shScr injected mice (p = 0.97). We next examined anxiety-like behavior in an open field test (Table 1). No differences were observed in the % time spent in the center of the open field between WT and *Lmo4gt/+* mice (p = 0.1) or in the total distance traveled (p = 0.07). Differences were also not observed in the amount of time spent in the center of the open field between shLmo4 and shScr injected mice (p = 0.764) as well as in total distance traveled (p = 0.44). Taken together, these results suggest that *Lmo4* functions in the BLC to specifically affect learned but not innate fear or anxiety.

**Table 1:** Reduction in *Lmo4* levels does not affect anxiety-like behavior.

Behavior	Parameter measured	WT	*Lmo4gt/+*	shLmo4	shScr
Elevated Plus Maze	% Open Arm Entries	41.14±3.03	40.41±2.23	36.54±3.5	37.66±3.76
	% Time in Open Arms	19.42±3.81	22.72±3.43		
	29.84±3.98	27.62±4.87			
Light/Dark Box	% Time in Lit Compartment	35.44±1.68	38.63±6.62	29.7±4.18	29.5±3.6
Open Field	% Time Spent in Center	16.77±1.85	12.74±1.44	9.92±1.47	10.56±1.48
	Distance Traveled (cm)	1028.2±61.81	817.91±90.89	1118.39±86.65	1031.17±68.48

The effects of global (*Lmo4gt/+*) or adult-and brain region-specific knockdown of *Lmo4* in the BLC on anxiety-like behavior was examined. Mice were subjected to the elevated plus maze, light/dark box, or the open field test. Mean values ± SEM are depicted. N = 11–12 (WT) and 9–11 (*Lmo4gt/+*) mice and n = 9–10 for shLmo4 and 10 for ShScr injected mice.

## Discussion

Results outlined in this study demonstrate a novel role for the transcriptional regulator *Lmo4* in the BLC in fear learning. LMO4 is predominantly expressed in pyramidal projection neurons of the BLC whose function is critical for fear learning. Reducing *Lmo4* levels either globally through out development or in an adult- and brain region-specific manner in the BLC results in enhanced freezing to both the context and the cue in a FC paradigm. Furthermore, *Lmo4gt/+* mice displayed enhanced c-Fos activation in the BLC following fear learning suggesting that increased neuronal activation following CS/US integration may underlie the increased freezing response observed in these mice. Anxiety-like behavior was unaffected by global or brain-region specific knockdown of *Lmo4*. In summary, our results indicate a selective role for *Lmo4* in regulating learned fear but not unlearned fear.

Contextual FC is conventionally thought to be dependent on both the hippocampus and the BLC, whereas cued FC is believed to be dependent on the BLC [34], [35]. *Lmo4gt/+* mice freeze significantly more to both the context and the cue. Since *Lmo4* is expressed in both the hippocampus and the BLC, we sought to determine the brain region within which *Lmo4* might be functioning to modulate fear learning. shRNA-mediated downregulation of *Lmo4* in the BLC recapitulated the phenotype observed in *Lmo4gt/+* mice whereas knockdown of *Lmo4* in the DG, a brain region known to be important for modulation of contextual fear learning [30], [31], had no effect on either contextual or cued FC. These results provide evidence for brain region specificity to the effects of *Lmo4* on fear learning. *Lmo4* is also highly expressed in the prefrontal cortex (PFC), and the CA1, and CA3 regions of the hippocampus, which are also implicated in certain aspects of fear learning [36], [37]. Our results suggest that knockdown of *Lmo4* in the BLC but not the DG is sufficient to recapitulate the behavioral phenotype observed in *Lmo4gt/+* mice. However, it is possible that *Lmo4* expression in other brain regions such as the PFC could also impact fear learning.

A role for transcriptional regulators in fear learning has been demonstrated previously. Knockdown of the transcriptional repressor methyl-CpG binding protein 2 (MeCP2) in the BLC has been shown to decrease freezing to the cue and results in a trend towards decreased freezing to the context [10]. Mice over expressing histone deacetylase 2 (HDAC2), which can repress transcription through histone deactivation, display reduced freezing to both the context and the cue [38]. Localized over expression of the transcription factor CREB in the BLC has been shown to enhance fear memory [9]. These results highlight the importance of gene expression-dependent changes in synaptic structure and function in the formation of fear memories. Further, LMO4 has been shown to associate with HDAC2 (in breast cancer cell lines, [21], [39]) and with CREB [16]. Hence, it is possible that LMO4 mediates some of its effects on fear learning through its interactions with other known regulators of learning and memory.

Studies have implicated a role for BLC in modulating unlearned fear/anxiety as well [32], [33]. Enhancement of glutamatergic neurotransmission in the BLC is thought to increase anxiety [33]. Since LMO4 is predominantly expressed in glutamatergic projection neurons of the BLC, we sought to determine whether reduction in *Lmo4* levels had an impact on anxiety-like behavior. We subjected *Lmo4gt/+* mice and their WT littermates as well as mice injected with shLmo4 and shScr to a battery of behavioral paradigms that measure anxiety-like behavior namely, the elevated plus maze, the light/dark box, as well as the open field test. We found no differences in anxiety-like behavior between WT and *Lmo4gt/+* mice. Several studies suggest a role for brain regions such as the PFC and hippocampus in anxiety-like behavior [40]. Our results with *Lmo4gt/+* mice suggest that reductions in *Lmo4* levels in several brain regions implicated in unlearned fear such as the hippocampus, PFC, and amygdala do not affect anxiety-like behavior. ShRNA-mediated knockdown of *Lmo4* in the BLC in adults also did not alter anxiety-like behavior. Taken together, these results argue against a role for *Lmo4* in anxiety like-behavior. However, an important caveat to these experiments is that the same set of mice was used in all three behavioral paradigms used to measure anxiety-like behavior. One of the advantages in subjecting the same mice to a battery of behavioral paradigms is the ability to fully explore the range of the phenotype [41], [42]. However, there are also potential confounds. Chief among them being that prior exposure to an anxiety-related test could influence behavior in subsequent tests [41], [42]. To minimize the effects of repeated testing, we allowed for the mice to recover for 1 week between tests. It is however possible that knockdown of *Lmo4* globally or in the BLC may lead to very subtle effects on anxiety-like behavior which we were unable to detect due to repeated testing. The lack of an effect of *Lmo4* downregulation in the BLC on unlearned fear is in contrast to its effects on learned fear. This not only argues for *Lmo4* functioning in specific pathways within the BLC but also suggests that knockdown of *Lmo4* levels does not result in a general disruption of BLC function.

Integration of CS and US in the lateral amygdala is thought to lead to long term potentiation and subsequent activation of signaling events leading to protein synthesis-dependent changes in synaptic structure and function [43], [44]. Indeed, fear memory has been shown to be sensitive to disruption by protein synthesis inhibitors administered immediately after training [45], [46]. Hence, we examined neuronal activation by quantifying c-Fos positive puncta in *Lmo4gt/+* mice and their WT littermates after FC. Our results indicate increased neuronal activation in the BLC of *Lmo4gt/+* mice after FC. This increased neuronal activation, especially in pyramidal projection neurons, may lead to enhanced consolidation of fear memories thereby resulting in the excessive freezing phenotype observed in *Lmo4gt/+* mice. We also found that pyramidal projection neurons, which are the primary loci for integration of CS and US in the BLC, are predominantly activated by fear learning in both WT and *Lmo4gt/+* mice. Furthermore, the ratio of pyramidal projection neurons to local interneurons that are activated by fear learning was unchanged in *Lmo4gt/+* mice in comparison to WT mice. Hence, it is likely that the increase in c-Fos positive puncta observed in *Lmo4gt/+* mice after fear conditioning results from activation of both pyramidal projection neurons and local interneurons, thereby leaving the ratio of activated cell types relatively unchanged. We did not observe any genotypic differences in the number of c-Fos positive puncta under control conditions suggesting that the BLA in *Lmo4gt/+* mice was not intrinsically more excitable than that of WT mice. In summary, our results suggest that reducing *Lmo4* levels in the BLC increases the number of neurons that are activated by fear learning. Reducing *Lmo4* levels may lower the threshold for neuronal activation by altering the expression of ion channels and consequently membrane conductance. Future experiments that compare the electrophysiological characteristics of principal neurons in the BLC of WT and *Lmo4gt/+* mice should provide insight into the underlying mechanisms. *Lmo4* levels are reduced in all pyramidal neurons in the BLC of *Lmo4gt/+* mice; yet, only a subset of these neurons are activated by coincident CS-US input. We hypothesize that the transcriptional targets/binding partners of *Lmo4* are different in subsets of pyramidal neurons in the BLC and this in turn determines which neurons are activated by fear learning.

Our results implicate a novel and specific role for *Lmo4* in the BLC in fear learning. LMO4 can function as an activity-dependent modulator of synaptic plasticity [24], [47]. Hence, it is possible that synaptic plasticity caused by CS/US integration may lead to *Lmo4*-dependent changes in gene expression. These changes in gene expression would normally function to dampen fear memory formation since reduction in *Lmo4* levels leads to enhanced freezing. Based on our results, we hypothesize that an LMO4-dependent transcriptional network is involved in fear learning. Future studies will utilize whole-genome approaches to identify transcriptional targets of LMO4 in the BLC, which may not only illuminate the mechanism by which LMO4 regulates fear learning, but also identify potential therapeutic candidates for treating cognitive impairments associated with a variety of neurological disorders.

## Supporting Information

Figure S1 **LMO4 expression is excluded from three of the major interneuron populations in the BLC.** LMO4 expression does not co-localize with markers for three of the major interneuron populations in the BLC namely calbindin (A), parvalbumin (B), and calretinin (C), suggesting that LMO4 expression is localized to pyramidal projection neurons of the BLC (see Figure 1). Scale bar: 50 µm.
(TIF)

Figure S2 **C-Fos is predominantly expressed in pyramidal projection neurons of the BLC in both WT and** ***Lmo4gt/+*** **mice after fear conditioning.** A) Majority of the c-Fos positive cells are also CaMKII-α positive (arrows) in both WT (top panel) and *Lmo4gt/+* mice (bottom panel). B) A representative image of a c-Fos positive and CaMKII-α negative cell (arrow) from a WT mouse is shown. Scale bars: 50 µm.
(TIF)
