# Supplementary figures and images for: Lmo4 in the Basolateral Complex of the Amygdala Modulates Fear Learning
Source: PLoS One. 2012 Apr 3;7(4):e34559. doi: 10.1371/journal.pone.0034559 (PMC3317997; doi:10.1371/journal.pone.0034559)

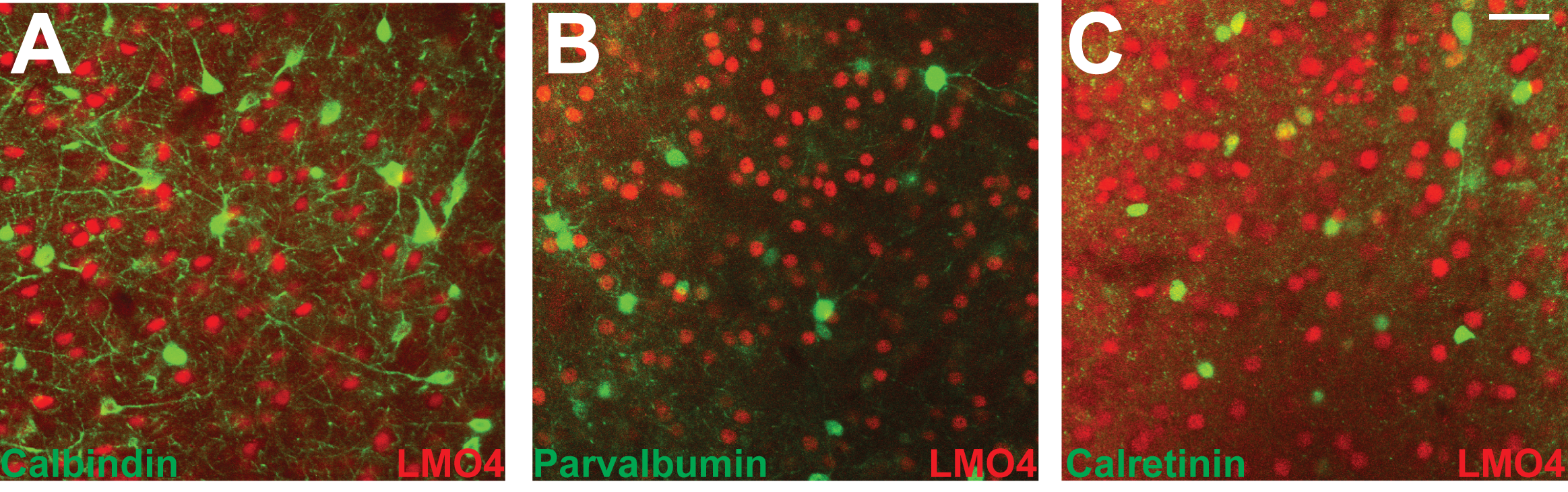

Supplement: Figure S1 — LMO4 expression is excluded from three of the major interneuron populations in the BLC. LMO4 expression does not co-localize with markers for three of the major interneuron populations in the BLC namely calbindin (A), parvalbumin (B), and calretinin (C), suggesting that LMO4 expression is localized to pyramidal projection neurons of the BLC (see Figure 1). Scale bar: 50 µm. (TIF) [file pone.0034559.s001.tif]

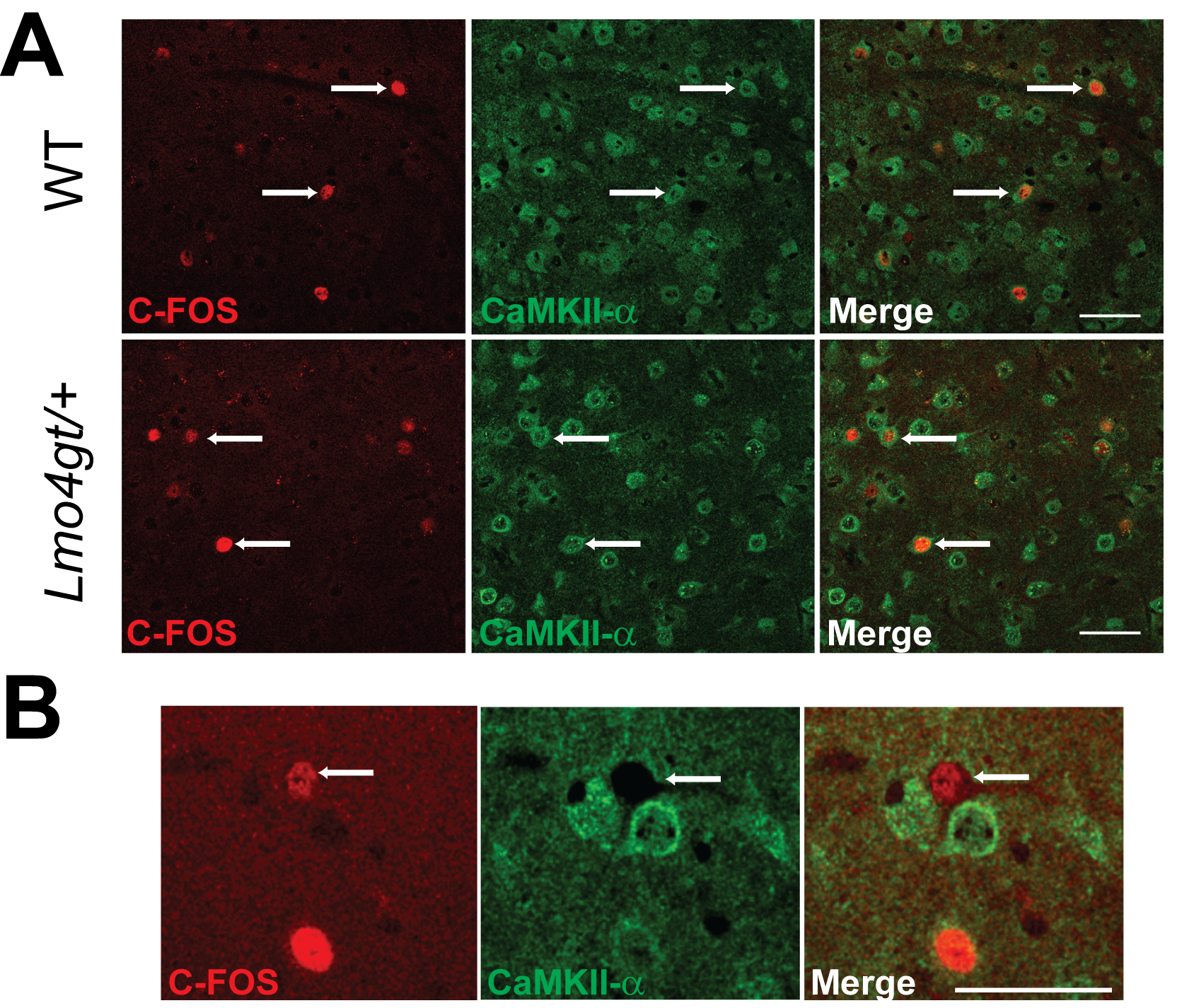

Supplement: Figure S2 — C-Fos is predominantly expressed in pyramidal projection neurons of the BLC in both WT and Lmo4gt/+ mice after fear conditioning. A) Majority of the c-Fos positive cells are also CaMKII-α positive (arrows) in both WT (top panel) and Lmo4gt/+ mice (bottom panel). B) A representative image of a c-Fos positive and CaMKII-α negative cell (arrow) from a WT mouse is shown. Scale bars: 50 µm. (TIF) [file pone.0034559.s002.tif]
